# Towards a better estimation of prevalence of female genital mutilation in the European Union: a situation analysis

**DOI:** 10.1186/s12978-020-00947-2

**Published:** 2020-07-08

**Authors:** L. De Schrijver, L. Van Baelen, N. Van Eekert, E. Leye

**Affiliations:** 1 International Centre for Reproductive Health, Ghent University, Corneel Heymanslaan 10, 9000 Ghent, Belgium; 2 Department of Public Health and Surveillance, Sciensano, Rue Juliette Wytsmanstraat, 14, 1050 Brussels, Belgium; 3 Centre for Population, Family & Health, University of Antwerp, Antwerp, Belgium

**Keywords:** Female genital mutilation, Female genital cutting, Female circumcision, Migrant health, Prevalence, European Union, Situation analysis

## Abstract

**Background:**

Female genital mutilation (FGM) is a harmful cultural practice that is predominantly documented in Africa, but also occurs in other parts of the world. Due to migration, women who have undergone FGM can also be found in the European Union (EU). Due to a lack of systematic representative surveys on the topic in EU, the prevalence of FGM and the number of women and children subjected to the practice remains unknown. However, information on the magnitude of the problem in the EU is necessary for policy makers to design and track preventive measures and to determine resource allocation.

**Methods:**

Between March 2015 and May 2015, we performed a situation analysis consisting of a critical interpretive synthesis and SWOT-analysis of available at the time peer reviewed and grey literature document on national prevalence studies on FGM in the EU. Studies estimating the prevalence of FGM and the number of girls and women subjected to the practice in the EU were mapped to analyse their methodologies and identify their Strengths, Weakness, Opportunities and Threats (SWOT). Distinction was made between direct and indirect estimation methods.

**Results:**

Thirteen publications matched the prioritized inclusion criteria. The situation analysis showed that both direct and indirect methodologies were used to estimate FGM prevalence and the number of girls and women subjected to FGM in the EU. The SWOT-analysis indicated that due to the large variations in the targeted population and the available secondary information in EU Member States, one single estimation method is not applicable in all Member States.

**Conclusions:**

We suggest a twofold method for estimating the number of girls and women who have undergone fgm in the EU. For countries with a low expected prevalence of women who have undergone fgm, the indirect method will provide a good enough estimation of the FGM prevalence. The extrapolation-of-fgm-countries-prevalence-data-method, based on the documented FGM prevalence numbers in DHS and MICS surveys, can be used for indirect estimations of girls and women subjected to FGM in the eu. For countries with a high expected prevalence of FGM in the EU Member State, we recommend to combine both a direct estimation method (e.g. in the form of a survey conducted in the target population) and an indirect estimation method and to use a sample design as developed by the FGM-PREV project. The choice for a direct or indirect method will ultimately depend on available financial means and the purpose for the estimation.

## Plain English summary

Female genital mutilation (FGM) is a harmful cultural practice that is predominantly documented in Africa, but also takes place in other parts of the world. Due to migration, women who have undergone FGM can also be found in the EU. Due to a lack of systematic representative surveys on the topic in the European Union (EU), the prevalence of FGM and the number of women and girls subjected to the practice in the EU remains unknown. However, information on the magnitude of the problem is necessary for policy makers to design and track preventive measures and to determine resource allocation. The main objective of this study was to analyse the methods used to estimate FGM prevalence and the number of girls and women subjected to this practice in the EU to date. Based on the identified strengths, weaknesses, opportunities and threats of the different approaches, we suggest a twofold method for estimating the number of women who have undergone FGM in the EU. For countries with a low expected prevalence of women who have undergone fgm, we expect that the indirect method will provide a sufficient estimation of the FGM prevalence. For countries with a high expected prevalence of FGM in the Member State, we recommend to combine both a direct and indirect estimation method and to use a sample design as developed by the FGM-PREV project. The choice for a direct or indirect method will ultimately depend on available financial means and the purpose for the estimation.

## Background

Female genital mutilation [FGM] is a practice that involves all procedures to the female genitalia for non-medical reasons. For many women, FGM is a traumatic experience with physical, psychological and sexual consequences [1–14]. According to UNICEF, an estimated 200 million girls and women have undergone FGM worldwide [15]. Growing evidence indicates that the practice is not only widespread in Africa, where it is predominantly observed [15], but also in parts of Asia - e.g. in Iran [16], Thailand [17], Indonesia [15] and the Middle East [18], suggesting that this number might be an underestimation.

Until 2015, FGM had been well documented in 29 countries in Africa and the Middle-East^1^ [15]: Benin, Burkina Faso, Cameroon, Central African Republic, Chad, Djibouti, Egypt, Eritrea, Ethiopia, Gambia, Ghana, Guinea, Guinea-Bissau, Iraq, Ivory Coast, Kenya, Liberia, Mali, Mauretania, Niger, Nigeria, Senegal, Sierra Leone, Somalia, Sudan,^2^ Tanzania, Togo, Uganda, and Yemen. The prevalence had been measured using a standard survey method developed by the Demographic Health Survey (DHS), published by MACRO, or the Multiple Indicator Cluster Surveys (MICS), published by UNICEF. In other countries there was only anecdotal evidence as was the case for Colombia [19], United Arab Emirates [11, 20], Oman [21], Brunei [22], Iran [16], Malaysia [23], Israel [24], Congo [25], and Thailand [17].

In the European Union (EU), the prevalence of FGM and the number of women and girls subjected to FGM are unknown. Prevalence is the proportion of individuals in a population with a certain characteristic, in this case having undergone FGM. When writing about estimated FGM prevalence in the EU, the population refers to the migrant population considered when estimating FGM prevalence within European host countries. FGM prevalence in FGM countries refers to the prevalence of FGM in practicing countries in Africa, Asia or the Middle East. The number of girls and women subjected to FGM in EU Member States refers to the total amount. Whilst FGM prevalence is interesting when looking at trends in the practice, the total number of girls and women subjected to FGM shows us the real scope of the existence of FGM. According to the European Institute for Gender Equality (EIGE) [26], there are no ongoing, systematic, representative surveys in the EU Member States which are similar to the DHS and MICS surveys and that use a harmonised approach to estimate FGM prevalence and the number of girls and women subjected to this practice [26].

However, with the increasing number of girls and women migrating from countries where FGM is practiced to the EU, the practice will remain a concern in the near future [27]. Providing information on the extent of FGM within the EU is important as a means to track progress on FGM prevention, to inform decision-makers and to determine resource allocation. By order of the European Commission through the DAPHNE program, the FGM-PREV study (2015–2017) [28] aimed to develop a common methodology to estimate the number of girls and women subjected to FGM and the prevalence of FGM in the EU. This paper provides an overview of in 2015 existing studies in the EU estimating the number of girls and women affect by FGM and its prevalence and discusses its methodologies within a SWOT-analysis.

## Methods

The study presented in this paper was part of a larger EU-funded project: *Towards a better estimation of prevalence of female genital mutilation in the European Union* (*FGM-PREV*) (JUST/2013/DAP/AG/5636) (November 2014 to March 2017). The ultimate aim of this project was to develop a common methodology and minimum standards for prevalence estimates of FGM in the EU, in order to generate comparable data. The first step of this project was a situation analysis including the SWOT-analysis discussed in this article (November 2014 to July 2015). The results of this SWOT-analysis functioned as the building stone for the development of the methodology to assess FGM prevalence and number of girls and women subjected to the practice in the EU. More information on *FGM-PREV* project can be found in the project report.^3^

The situation analysis consisted of a critical interpretive synthesis of available at the time peer reviewed and grey literature documents on national numbers of girls and women subjected to FGM and/or FGM prevalence in the EU. The inclusion criteria were the following: any documents on estimating FGM prevalence and/or the number of girls and women subjected to the practice in the EU, and on numbers of girls and women originating from the 29 countries where FGM has been well documented up to July 2015 who had migrated to the EU and who have undergone FGM. The focus was on documents that were published after 2000. Only articles and reports about estimation of FGM prevalence and/or the number of girls and women subjected to FGM on a national level in a specific member state or in the EU were selected. Excluded from this synthesis were: articles published in popular media such as newspapers or magazines, articles we did not have full access to, regional studies, documents that were published before 2000 or did not concern the eu.

Firstly, all relevant information on data available on the prevalence of FGM in the eu has been collected through a systematic web-based search. This desk research was performed in English with key terms such as ‘female genital mutilation’, ‘FGM’, ‘female genital cutting’ and ‘female circumcision’, combined with ‘prevalence’ and the 28 different EU member states, ‘European union’, ‘EU’, ‘western countries’ and ‘European member states’. They were introduced in well-established academic and scientific databases, such as Google Scholar, Web of Science, and PubMed. Further, Sociological Abstracts, Social Science Research Network, Heinonline, and EBSCO have also been searched but these databases did not generate new references.

Secondly, the EIGE Country Reports [29] were taken into account. These publications from 2013 present national reports on FGM, covering the eu-27 and Croatia [29]. From this study, articles were selected if the title and abstract had a specific reference to prevalence of FGM on a national level, published from 2000 onwards, and that had not been found through the web-based search (these are references [30–33]).

Finally, we also contacted key persons and institutions, by e-mail or by phone. Some of the prevalence studies that have been carried out, were not written in English. If one of the authors was familiar with the language, as was the case with French and Dutch (e.g. the Belgian study by Dubourg & Richard, 2014 [34]), the reports were directly analysed; if not, we contacted the researchers and asked them for an extensive English summary. Through these contacts we had access to Italian (see Farina, 2010 [35]; Ortensi, Farina & Menonna, 2015 [36]; Farina & Ortensi, 2015 [37]), Spanish (see Kaplan & Lopez, 2013 [38]), and German studies (see Terre des Femmes, 2015 [39]).

The literature search took place from the beginning of March 2015 until the middle of May 2015. The titles and abstracts of the retrieved records were screened for relevance to FGM prevalence. Twenty-nine papers were included for further analysis [30, 31, 34–37, 39–58]. From these 29 documents, 20 articles were identified as studies estimating FGM prevalence and/or number of girls and women affect by FGM in the EU.

**Fig. 1:**
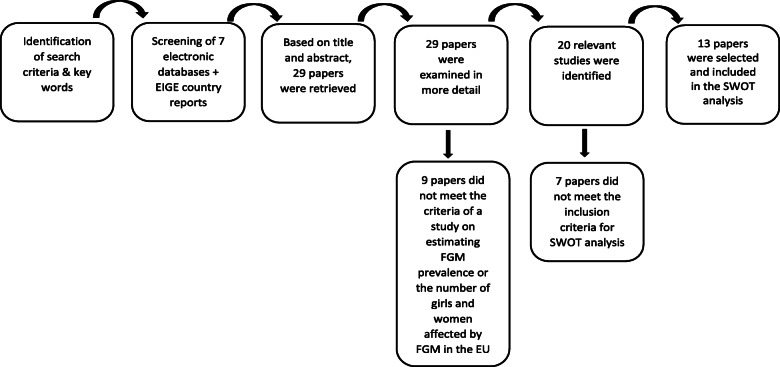
Schematic overview of the literature review process

In order to perform the SWOT-analysis, the inclusion criteria were defined more narrowly. Only publications about FGM prevalence and/or number of girls and women subjected to FGM in the EU were selected if they concerned prevalence estimations at a national level. Since the specific objective of this study was to define a common methodology that could be utilized on a EU level, it was decided to exclude regional studies because of the scale of the research. This led to a final inclusion of 13 publications [30, 31, 34–37, 39–45]. The literature selection algorithm is presented in Fig. 1. Table 1 gives a summary of the selected papers.

**Table 1:** Summary of literature included in review

Reference	Context	Year	Method	Sample	Aim	Findings
1.Dorkenoo, E., Morison L., & Macfarlane, A. (2007) [40]. A statistical study to estimate the prevalence of female genital mutilation in England & wales: summary report. London (UK): Forward	England & Wales	2007	Extrapolation-of-fgm-countries-prevalence-data-method: DHS & MICS applied to Census and birth registration data from the Office for National Statistics for England and Wales (Indirect) Estimations corrected for: - age - ethnicity - religion	174.528 women resident in England or Wales in 2001, who were born in an FGM practising country (Census)	- To estimate the number and girls affect by FGM and the prevalence of FGM in England and Wales among women aged 15 and over, the number of registered maternities to women who have undergone FGM and the number of girls below the age of 15 at risk for FGM & the type of FGM	Nearly 66.000 women with FGM were living in England or Wales in 2001
2.Andro, A., Lesclingand, M., Cambois, E., & Cirbeau, C. (2009) [30]. Excision et Handicap (ExH): Mesure des lésions et traumatismes et évaluation des besoins en chirurgie réparatrice. Paris: INED.	France	2009	Survey: Etude de L’histoire Familiale. Both quantitative and qualitative data was gathered about FGM prevalence in French speaking migrants or those born from migrant parents in a multicentric study in a medical setting (Direct) Extrapolation-of-FGM-countries-prevalence-data-method: prevalence data observed in the countries of origin were applied to the numbers of each subgroup (Indirect)	380.000 men and women resident in France in 1999, who were born in an FGM practising country	- To estimate the number of girls and women affect by FGM and FGM prevalence on a national level - To understand the family dynamics around the practice of FGM and the migration situation - To estimate quantitatively het consequences of FGM for these women	Nearly 53.000 adult women with FGM were living in France in 2004. The distribution of these women in France is unequally dispersed.
3.Farina, P. (ed.) (2010) [35]. Indagine sulla presenza nel territorio lombardo di popolazione a rischio in relazione alla salute sessuale e riproduttiva e alle mutilazioni genitali femminili. Milano: Istituto Regionale di Ricerca della Lombardia.	Italy	2010	A general survey on sexual and reproductive health using facility-based and respondent driven sampling (direct) The survey can be compared to DHS and MICS data about FGM in the countries of origin.	2011 immigrants aged 15–49, living in Lombardy (Italy) in 2010	- To estimate the number and prevalence of women aged 15–49 living in Lombardy (Italy) subjected to FGM - To map sexual and reproductive health among immigrants in Italy in 2010	In Lombardy (Italy) an estimated 17,2% of African immigrant women have undergone FGM, which is less than 5% of all immigrants. 1500 girls are at risk of FGM.
4.Dubourg, D., Richard, F., Leye, E., Ndame, S., Rommens, T., & Maes, S. (2011) [41]. Estimating the number of women with female genital mutilation in Belgium. The European Journal of Contraception and Reproductive Health Care, 16(4):248–57.	Belgium	2011	Extrapolation-of-fgm-countries-prevalence-data-method: MICS & DHS data applied to 1st and 2nd generation female migrants with estimates based on Federal Directorate General of Statistics, the Federal Agency for the Reception of Asylum Seekers (Fedasil), the Office de la Naissance et de l’Enfance (ONE) and Kind & Gezin (G&G) (indirect) Estimations corrected for: - age	All immigrant women from countries where FGM is practised and their daughters born in Belgium since 1998.	To estimate in Belgium: - the number of women with FGM - the number of girls at risk of FGM - the target population of medical and social services	Amongst the 22.840 women & girls living in Belgium, origination from an FGM practising country, 6260 have ‘most probably undergone’ FGM and 1975 girls were at risk of FGM. The distribution of these women in Belgium is unequally dispersed.
5.Korfker, D.G., Reis, R., Rijnders, M.E.B., Meijer-van Asperen, S., Read, L., Sanjuan, M. et al. (2012) [42]. The lower prevalence of female genital mutilation in the Netherlands: a nationwide study in Dutch midwifery practices. International Journal of Public Health, 57: 413–420.	The Netherlands	2012	Extrapolation-of -prevalence-data-method based on a retrospective nationwide survey of midwives (indirect)	All 513 midwifery practices in the Netherlands were invited. The response rate was 93% (*n* = 478).	- To estimate the FGM prevalence and number of girls and women subjected to FGM giving birth in 2008 in the Netherlands.	Retrospective reports by midwives led to a number of 470 women (0,32%) with a history of FGM who gave birth in 2008 in the Netherlands.
6.Köszeghy, L. (2012) [31]. FGM prevalence in Hungary-estimation. Retrieved March 26, 2015, from: http://mona-alapitvany.hu/wp-content/uploads/2012/11/fgm-prevalence-in-hungary-estimation.pdf	Hungary	2012	Extrapolation-of-fgm-countries-prevalence-data-method: applied to all women with all kinds of permits coming from FGM-countries (indirect)	All women from countries where FGM is prevalent: - with a residence permit - those who obtained a refugee status in the past 10 years - those who obtained temporarily protected status - currently residing in one of the reception centres	- To estimate the number of girls and women affect by FGM and FGM prevalence in Hungary	The number of migrant women subjected to FGM in Hungary is estimated to lie between 170 and 350. There is an unequal distribution of these women in different Hungarian areas.
7.Exterkate, M. (2013) [43]. Female Genital Mutilation in the Netherlands. Prevalence, incidence and determinants. Utrecht: Pharos Centre of Expertise on Health for Migrants and Refugees	The Netherlands	2013	Extrapolation-of-fgm-countries-prevalence-data-method: Data from national representative surveys in the country of origin were used: DHS, MICS. These data were combined with data from registers from the Dutch Central Statistical Office, Central Agency for the Reception of Asylum Seekers, Youth Health Care and Reporting Centres of Child Abuse and Neglect. (Indirect) Estimations corrected for: - age - region of origin - religion Qualitative contextual information was also gathered through focus group discussions.	Women living in the Netherlands in 2012 and originating from countries where FGM is traditionally practised.	- To estimate the prevalence of FGM and number of girls and women at risk of FGM in the Netherlands	The total number of women with FGM is estimated on 29.120 women in the Netherlands. From almost 70.000 women (1% of the Dutch female population) origination from FGM practising countries, nearly 40% have undergone FGM.
8.Dubourg, D., & Richard, F. (2014) [34]. Studie over de prevalentie van en het risico op vrouwelijke genitale verminking in België. Brussel: FOD Volksgezondheid.	Belgium	2014	Extrapolation-of-fgm-countries-prevalence-data-method: estimation 1st & 2nd generation (based on ADSEI, data on asylum seekers, birth registration), extrapolation of this prevalence (per age category) in country of origin (DHS & MICS) on women & girls living in Belgium (indirect) Estimations corrected for: - age	All immigrant women from countries where FGM is practised and their daughters born in Belgium since 1998.	To update the data from 2008 on the prevalence of FGM in Belgium: - the number of women with FGM - the number of girls at risk - the target population of medical and social services	Among the 48.092 girls and women living in Belgium on 31 December 2012, originating from a country where FGM is practised and their daughters born in Belgium, it was estimated that 13.112 of them ‘had probably already undergone FGM’ and that 4084 were ‘potentially at risk of FGM’.
9.Macfarlane, A., & Dorkenoo, E. (2014) [44]. Female genital mutilation in England & Wales: Updated statistical estimates of the affected women living in England and Wales and girls at risk. Interim report on provisional estimates. London: City University London & Equality Now.	United Kingdom	2014	Extrapolation-of-fgm-countries-prevalence-data-method: DHS, MICS, Census 2011, birth registration women born in FGM practicing countries, but who are member of a non-practicing FGM population were excluded (indirect) Estimations corrected for: - age - ethnicity - religion - prevalence in FGM practicing countries	Women who were born in countries where FGM is traditionally practised and data on FGM is available. Women belonging to populations or religions that do not practise FGM were excluded.	- To update the estimation of the prevalence of FGM in England and Wales from 2007. - To produce updated and reliable estimates of the prevalence of women born in FGM practicing countries, the number of women with FGM living in England and Wales as a whole and in each local authority area. - To disseminate these estimations to local authorities to enable them to provide guidance in the reduction of FGM in their area.	In 2011, an estimated 137.000 girls and women with FGM were permanent residents in England and Wales. There were 103.000 women aged 15–49; 24.000 were 50 and over; and nearly 10.000 girls aged 0–14 were at risk of FGM.
10.Baillot, H., Murray, N., Connelly, E., & Howard, N. (2014) [45]. Tackling Female Genital Mutilation in Scotland. A Scottish model of intervention. Glasgow: Scottish Refugee Council, London School of Hygiene & Tropical Medicine	United Kingdom	2014	Extrapolation-of-fgm-countries-prevalence-data-method: Examination of available secondary data (2011 Census, birth registration data 1993–2012, 2013 Pupil Census) and administrative records from stakeholders on the FGM strategic were used too (indirect)	Men, women and children living in Scotland and origination from one of 29 FGM-practising countries identified by UNICEF in 2013.	- To identify populations which are potentially subjected to FGM and to explore interventions across the European Union in order to present data and recommendations for the development of a Scottish model of intervention.	In 2011, 23.979 men, women and children, origination from FGM practising countries, were living in Scotland. Potentially affected communities can be found in every local authority area in Scotland.
11.Farina, P., & Ortensi, L. (2015). Improving estimates of the prevalence of Female Genital Cutting among migrants in Western countries. Retrieved March 26, 2015, from: http://paa2015.princeton.edu/uploads/152685	Italy	2015	Extrapolation-of-fgm-countries-prevalence-data-method: DHS, MICS (indirect) Estimations corrected for: - age - selectivity of migration - region of origin - prevalence in FGM practicing countries Survey: Migrants’ Selection Hypothesis is used to correct national prevalence estimates. This correction was calculated on the prevalence variations among different socio-demographic groups and inter-regional variations in the migrants’ countries of origin + assessment of the reliability of this correction (comparison with direct estimates)	Women aged 15–49, born in FGM practising countries and living in Italy, more specific in the region of Lombardy. All women were selected irrespective of current nationality or legal status.	- To present a new method to estimate the prevalence of FGM among first generation migrants.	The selection Hypothesis corrects the prevalence estimation among immigrants in Lombardy, Italy to a lower number than expected based on the prevalence in their respective practising country.
12.Ortensi, L., Farina, P., & Menonna, A. (2015) [36]. Improving estimates of the prevalence of Female Genital Mutilation/Cutting among migrants in Western countries. Demographic Research, 32: 543–562.	Italy	2015	Extrapolation-of-fgm-countries-prevalence-data-method: DHS, MICS (indirect) Estimations corrected for: - age - selectivity of migration - region of origin - prevalence in FGM practicing countries Survey: Migrants’ Selection Hypothesis is used to correct national estimates of prevalence and to obtain a better estimation of the prevalence of FGM among overseas communities (comparison with direct estimates)	Women aged 15–49, born in FGM practising countries and living in Lombardy (Italy). All women were selected irrespective of current nationality or legal status.	- To present an improved method of indirect estimation of the prevalence of FGM among first generation migrants based on a migrant selection hypothesis. - To provide a criterion to assess the reliability of indirect estimates.	The Selection Hypothesis leads to a prediction of a lower prevalence of FGM than can be expected based on the prevalence in the country of origin.
13.Terre des Femmes (2015). Dunkelzifferstatistik zur weiblichen Genitalverstümmelung in Deutschland. Retrieved March 26, 2015, from: http://www.frauenrechte.de/online/index.php/themen-und-aktionen/weibliche-genitalverstuemmelung2/aktuelles	Germany	2015	Extrapolation-of-fgm-countries-prevalence-data-method: DHS, MICS. Effects of migration were also taken into account. Estimations corrected for: - age (2013 + 2015) - migrant generation (2015)	Women living in Germany, originating from countries where FGM is practiced (Africa & Asia).	- To estimate FGM prevalence in Germany - To show that FGM prevalence is reduced by migration	An estimated 5956 women at risk of FGM and 35.715 women who underwent FGM, are living in Germany. These numbers are lower than estimated in the countries of origin

Once the selection of 13 relevant articles was made, a SWOT-analysis was conducted. Studies estimating the number of girls and women subjected to FGM and FGM prevalence in the EU were mapped to identify Strengths, Weakness, Opportunities and Threats (SWOT) of their FGM estimation methods. Strengths and weaknesses are focusing on internal factors, opportunities and threats are external factors [59, 60]. Distinction was made between indirect and direct estimation methods. Indirect estimation methods use secondary data sources on both FGM prevalence in practicing countries and the absolute number of women originating from countries where FGM is practiced to estimate the number of girls and women subjected to FGM in EU-countries. FGM prevalence in the EU is then calculated based on this estimated number of girls and women subjected to FGM in the EU. Secondary data sources on the number of girls and women originating from a country where FGM is practiced are population registers, birth registers, registers of asylum seekers, results from national census or a combination of some of these data sources. Direct estimation methods on the other hand, require direct data collection in the population of interest. For example, by conducting a survey on FGM in the target population.

Finally, data from the national statistical offices from all EU countries, on variables that are essential for FGM prevalence estimations was requested to assess the applicability of different approaches that were suggested in previous studies throughout the EU. It mainly concerned information on variables such as age, nationality and ethnicity of women coming from countries where FGM is practiced, and their female descendants.

## Results

From the 28 national statistical offices, only 14 provided us with the requested information. This information showed that there is no standardized way for the presentation of data in the EU; definitions of demographic variables are quite different, and that categories of e.g. age are not uniform. As a result, it would be impossible to implement a uniform approach for the prevalence estimation of FGM in the EU based on these data sources alone. The SWOT-analysis of the methods used to estimate the prevalence of FGM and the number of girls and women subjected to it in the EU, gave more insight in how the calculations and estimations were done using these different presentations of data in the EU.

Nine out of thirteen papers referred to studies that estimated FGM prevalence and number of girls and women subjected to the practice in a specific EU member state in an indirect manner (see [31, 34, 39–45]). Four documents (see [30, 35–37]) referred to studies that additionally included direct estimations. All 13 documents included in the literature review, referred to studies that used quantitative methods to estimate the prevalence of FGM. In two documents, i.e. the study from France performed by Andro et al. in 2009 [30] and the study from Exterkate in the Netherlands from 2013 [43], qualitative data was also used.

### Indirect methods to estimate fgm prevalence and number of girls and women subjected to FGM in the EU

The majority of the studies (9 out of 13) estimated FGM prevalence and the number of girls and women subjected to FGM using only an indirect estimation method. These nine studies applied the so-called ‘extrapolation-of-fgm-countries-prevalence-data-method’.^4^ As mentioned before in the EIGE study [26], the extrapolation-of-fgm-countries-prevalence-data-method is a method whereby the FGM prevalence rate in the countries of origin (for girls and women age 15 to 49, as reported by the DHS and MICS) is multiplied by the total number of girls and women in the country of destination coming from or born to a mother originating from one of the countries where FGM is practiced. The estimation of the overall number of women who have undergone FGM in the country of destination is the sum of the estimated number of women who have undergone FGM for every country of origin. The multiplication of both gives the estimated number of girls and women subjected to FGM in the EU. By using indirect estimations, one thus does not calculate a prevalence directly, but use existing ones to apply them on the migrant populations in the EU. Estimated FGM prevalence in EU-countries can then be calculated based on the estimated number of girls and women who have undergone the practice. Only Korfker et al. [42] also used primary data from a retrospective nationwide survey of midwives who were asked about the total number of women with FGM they had under surveillance in 2008.

Discussing the extrapolation-of-fgm-countries-prevalence-data-method requires that one should pay specific attention to the specific migrant population considered based on secondary data and how FGM countries’ prevalence is applied onto this population– especially when calculating the estimated number of girls and women subjected to FGM.

The migrant population involved does vary between various studies. As there is no universally accepted definition of migrants, different choices on defining the migrant population can be made, e.g. based on citizenship [39], origin [26, 31, 34, 43], origin of mother [34, 39], ethnicity [44], nationality or place of birth [34, 40, 41, 43, 44], or place of birth mother [44]. Further, some studies look at legal status too and may include applicants for international protection, undocumented migrants and so on. Others do not. Additionally, this depends on the available secondary data on the migrant population. In the EU there is no uniform registration method making migrant register vary over countries. Secondary sources providing information on the migrant population included in these studies were population registers [31, 34, 39, 41], birth registers [34, 40–42, 44, 45], child protection register [43], registers of applicants for international protection [34, 41, 43], results from a national census [40, 44, 45] or a combination of some of these data sources.

FGM prevalence in FGM countries are retrieved from the DHS and MICS. A first indicator of national FGM is the proportion of girls and women of reproductive age (15 to 49) who have experienced any form of FGM. In the 29 countries where FGM is concentrated, almost all girls are cut before the age of 15 as this reflects their final FGM status. A second indicator of national prevalence measures the extent of cutting among daughters aged 0 to 14, as reported by their mothers. Prevalence data for girls reflect their current – not final – FGM status, as many of them may not have reached the customary age for cutting at the time of the survey [61].

The application of prevalence in FGM countries onto migrant populations in the EU varies. Whilst the basis calculation is to multiply the FGM prevalence rate in the countries of origin for girls and women age 15 to 49 by the total number of girls and women in the country of destination coming from or born to a mother originating from one of the countries where FGM is practiced, estimates may be applied using additional nuanced corrections based on theoretical arguments as well as the outcome of additional qualitative research.

The first type of corrections is based on the age of girls and women in the countries of origin and destination. In most DHS and MICS studies, FGM prevalence figures are given for the age group between 15 and 49 (with the exception of Egypt and Togo where prevalence for girls 0–14 is also known). However, the majority of the girls undergo FGM before the age of 15, with the median age for undergoing FGM in the 29 countries where FGM is documented ranging between 1 and 14. Dubourg et al. [41], Dorkenoo et al. [40], Dubourg and Richard [34], Macfarlan and Dorkenoo [44], Ortensia et al. [36] estimated the FGM prevalence for women aged over 50 by taking the FGM prevalence figures for the women aged 45–49 and applying these to the entire 50+ cohort. Likewise, the FGM prevalence figures for girls aged 15–19 were applied to the cohort younger than 15 years old (0–14). Exterkate [43] used the median age for undergoing FGM, as reported by DHS or MICS, to estimate the prevalence of FGM among girls 0–14, based on their age of arrival. To estimate the number of first-generation migrants who are cut, is assumed that girls or women who arrive after the median age of FGM are cut, and those who arrived before are not. For the second generation, it is assumed that daughters older than 12 have the same FGM prevalence as in the country of origin, and those younger than 15 are assumed to be uncut. Terre des femmes [39] calculated the risk to be cut per 5-years age group, pointing out the median age indicating the age where half of the girls and women are probably cut.

The second type of corrections considers regional differences regarding the practice of FGM in the countries of origin. Exterkate’s second correction relates to FGM prevalence figures per province or region, as given by the DHS and MICS [43]. In most countries of origin, the variation between the different regions is substantial. For example, in the southern region of Senegal 69,4% of women have undergone FGM, whereas only 6,3% of women in the central region have been subjected to FGM [62]. In Kenya the differences are even bigger: 0.8% of the women in the Western province have undergone FGM compared to 97,5% in the North-eastern province bordering Somalia [63]. Since the registration system for migrants in the Netherlands also encodes the region or province of origin, it was possible to apply regional, rather than national prevalence figures to estimate the prevalence of FGM in the Netherlands. Dorkenoo et al. [40] and Macfarlane and Dorkenoo [44] excluded members of the migrant population form the calculations when they were from an ethnicity of religious community were FGM was not practiced in the country of origin.

The third type of corrections adjust the estimation of FGM in the EU for varying occurrence of FGM in migrant population, stating that this differs from FGM prevalence in their country of origin. Various assumptions may have been made on the occurrence of FGM within the migrant population. Exterkate [43] nuanced for this matter in her study by estimating three variants (high, middle, low) of FGM occurrence in the migrant population, based on literature and focus group discussions with respondents selected through local NGO’s. This interval was a statistical estimation where the maximum meant that there was no influence of migration whatsoever (and the practice continued at the same rate in the EU as in the countries of origin) and the minimum meant that after migration the attitudes and behaviour towards FGM had completely changed (and nobody had changed FGM status after arrival in the country of destination). Exterkate [43] added qualitative data to give insight into dynamics of social pressure and reasons for (dis) continuity of the practice after migration. Based on the results of the focus groups, she argues that the ‘real’ prevalence is closer to the minimum than to the maximum, which means that some to most but not all migrants have changed their attitudes and behaviour towards FGM. Terres des femmes [39] corrected for the influence of migration by differentiating the occurrence for the first generation (FGM prevalence in EU 100% similar to FGM countries) and the second generation (FGM prevalence in EU 50% similar to FGM countries). Farina and Ortensi [37] and Ortensi et al. [36] corrected the FGM prevalence as presented by DHS and MICS based on migration characteristics such as age, wealth, education and level of urbanization of the migrants in their country of origin. They justified their corrections with the hypothesis that migration is a selective process, the so-called ‘selection hypothesis’. In addition, Ortensi et al. [36] also propose to correct the estimations based on less recent DHS and MICS data for younger generations. As the phenomenon is changing in the country of origin due to e.g. prevention campaigns, younger generations may be less likely to have be subjected to FGM. Older surveys may thus be less representative for younger than for older cohorts [36].

### Direct methods to estimate fgm prevalence and number of girls and women subjected to fgm in the EU

Among the analysed documents within this project, two described how they performed direct estimates: Andro et al. in 2009 [30] estimating FGM prevalence in France and Farina in 2010 [35] estimating FGM prevalence in Italy.

Andro et al. recruited respondents through gynaecological cabinets whereas the Italian publications [35–37] reported on a combination of facility-based and snowball sampling. Samples could not be randomized because the number of women who have undergone FGM is very low in comparison with the general population. The European Parliament Resolution of March 24th 2009 on FGM indicated that an estimated 500,000 women living in the EU have been subjected to FGM [64]. Since the EU has approximately 500 million inhabitants, this gives a proportion of 1/1000. Moreover, migrants are not equally distributed over the 28 EU member states, nor within the countries. Often, they are living in urban areas and even within these cities they tend to live only in certain neighbourhoods. This means that nationwide randomized samples to estimate FGM prevalence would be very inefficient and costly. Instead, both studies were based on a sample of women who were born in countries where FGM is practiced and the selection was done within a specific geographical area, i.e. a province or a region with a high number of migrants and through facilities that were frequented by women who might have undergone FGM. Based on the results of this survey, the estimated FGM prevalence was extrapolated and thus applied to the migrant population.‘.

In the studies from Farina and Ortensi [37] and Ortensi et al. in 2015 [36] indirect estimations as discussed above were compared to the direct estimations as calculated in the paper of Farina [35].

Farina [35] conducted a study directly estimating FGM prevalence in Lombardy. Collecting data on FGM was a module within a broader survey on sexual and reproductive health, similar to DHS surveys, in the migrant population in Lombardy. In total 2011 migrant women 14–49 years old were interviewed, from which half (1020) were coming from FGM practicing countries and thus questioned using the FGM module. The sampling was based on nationality and age-groups: “young” (15–24 years old), “adults” (25–39 years old) and “elderly” (40–49 years old). Since special attention ought to be given to FGM, sampling was organised such that sufficient respondents were from FGM practicing countries. Looking at DHS data of countries of origins, nationalities where FGM prevalence is over 2% were guaranteed to have at least 80 respondents in the Lombardy survey – having sufficient respondents in each age group. Respondents were asked about their FGM status and difference was made between the symbolic nick and cutting. As a result, it was possible to estimate FGM prevalence in the migrant population in Lombardy, per nationality for women at reproductive age (14–49 years old). Indirect estimates were found here to be accurate for countries where FGM is widespread. Finally, after comparing direct and indirect estimates, direct estimates were used when available, and the ‘extrapolation-of-fgm-countries-prevalence-data-method’ with corrections only for countries not included in the survey.

Different from Farina’s study, the principal objective of Andro’s study [30] was not to estimate FGM prevalence but rather to document the past and current situation of women who had undergone FGM and to compare them with women without FGM using a population based case-control study. The recruitment and interviews were conducted in 74 mother-and-child health centres and hospital departments providing gynaecological and family planning services in five French regions. Based on secondary data, the number of girls and women subjected to FGM was indirectly calculated by assuming that in each subgroup of girls and women from the same country of origin at risk, the proportion of girls and women with FGM was the same as in the country of origin. After applying the extrapolation-of-fgm-countries-prevalence-data-method, Andro et al. [30] calculated an interval based on three hypothesis: (a) only women who were born in the country of origin and who arrived after the age of 15, (b) only women who were born in the country of origin and (c) all women who originated from a country of origin where FGM is practiced. They conclude that the second (‘medium’) hypothesis is most likely to correspond with reality. Andro et al. [30] thus used the direct methodology using a survey to correct the initial indirect estimations of girls and women subjected to FGM in France.

## Discussion

### Strengths and limitations of indirect estimation methods

Until May 2015, most FGM prevalence figures in the EU were the result of a triangulation of European population data, European or national census data and data from DHS and MICS, the so-called ‘extrapolation-of-fgm-countries-prevalence-data-method’. This indirect estimation, with or without corrections, can be done regularly because the technique is cheap and not complex compared to direct estimations. At the same time, it allows policy makers to have a reliable approximation of the estimated number of girls and women who have undergone FGM and FGM prevalence, to look for trends and to evaluate the impact of, among others, prevention programs. However, there are several limitations to this indirect method.

Firstly, the indirect estimations are based on the collection of migration related data. Yet, defining a migrant and measures to be used when studying migrant-related issues, such as FGM, is problematic. The un [65] defined an international migrant as ‘any person who changes his or her country of usual residence’; a ‘long-term migrant’ is someone who moves to a country other than his/her usual residence for a period of at least a year and a ‘short-term migrant’ is a person who moves for at least 3 months. However, as Johnson [66] has stated about migration, ‘a primary problem is defining exactly what measure one should use to capture the population of interest: country of birth, citizenship, “race”, ethnicity, culture or migrant status.’ Other research (see e.g. Agyemang et al. [67] and Levecque et al. [68]) has mentioned that there is no consensus on appropriate terms for the scientific study of health by ethnicity and that there is no universally accepted definition of ‘migrant’. Many studies have focused on the diversity among immigrant populations (see e.g. Brimicombe [69] and Faist [70]). Vertovec [71] even referred to the variety of immigrant communities in Britain with the term ‘super-diversity’ because of the greater number of attributes such as age, gender, origin, language, religion etc. which makes it difficult to define a homogenous sample. Finally, there are different legal statuses by which these immigrants can be registered (incl. Undocumented migrants, applicants for international protection, adopted citizenship). A common approach in the EU to register these migrants in the same way is missing (cf. supra) and only a uniform registration in every EU member state would allow for a reliable estimation of women coming from countries where FGM is practiced.

Secondly, even with a uniform registration, the results of an indirect estimation will always lag behind the ‘real’ situation because the number of migrants in the EU fluctuates from year to year. For example, in Spain, Kaplan and López [38] found an increase of 40% of women originating from countries where FGM is practiced between 2008 and 2012, corresponding to an absolute increase of 16.361 women. Macfarlane and Dorkenoo [44] found in their analysis of the evolution in the uk between 2001 and 2011 that the total number of female migrants from the 29 countries where FGM is documented, had increased with more than 100,000 women in 10 years. This difference was not equally distributed among all nationalities. The number of women born in Kenya and living in the uk for example, decreased between 2001 and 2011 by almost one third (from 45,396 to 31,740) whereas the number of Somalians almost tripled (from 15,744 to 43,558). The estimated number of girls and women who have likely undergone FGM increased from 65,790 in 2001 to 137,000 in 2011 [44].

As shown in Table 2, not only in the EU but also in the countries of origin, FGM prevalence figures are fluctuating. The overall percentage of women who have undergone FGM has changed in most FGM practicing countries (see Table 2), as is the case for instance in Benin (a reduction of 9.5% of the women who report being subjected to FGM between 2001 and 2011/2012) or Egypt (a reduction of 6.2% between 1995 and 2008). Moreover, when comparing older and younger cohorts of girls and women, we see that in all 29 countries in Africa and the Middle East where FGM is documented, the youngest age cohort (15–19) having prevalence figures that are considerably lower than the oldest cohort (45–49). For example, the difference between both cohorts is 41.3% in Liberia [72], 34.2% in Kenya [73] and 31.6% in Burkina Faso [74]. However, it remains unclear what causes these changes. Only in some countries with very low prevalence figures such as Niger (− 2.5%), or Uganda (− 0.8%) and in some countries with very high prevalence figures, such as Sudan (− 1.2%) the difference between both cohorts is marginal.

**Table 2:** National % FGM prevalence (15–49) known in July 2015 in countries of origin according to DHS and MICS surveys

	Most recent (t)		t-1		t-2		t-3		t-4		Max-Min
Benin	2011/12	7,3%	2006	12,9%	2001	16,8%					9,5%
Burkina Faso	**2010**	75,8%	**2006**	72,5%	**2003**	76,6%	**1998/99**	71,6%			5,0%
Cameroon	**2004**	1,4%									0,0%
Central African Republic	**2010**	24,2%	**2006**	25,7%	**1994/95**	43,4%					19,2%
Chad	**2010**	44,2%	**2004**	44,9%							0,7%
Côte d’Ivoire	**2011/12**	38,2%	**2006**	36,4%	**2005**	41,7%	**1998/99**	44,5%	**1994**	42,7%	8,1%
Djibouti	**2006**	93,1%									0,0%
Egypt	**2008**	91,1%	**2005**	95,8%	**2003**	97,0%	**2000**	97,3%	**1995**	97,0%	6,2%
Eritrea	**2010**^**a**^	83,0%	**2002**	88,7%	**1995**	94,5%					11,5%
Ethiopia	**2005**	74,3%	**2000**	79,9%							5,6%
Gambia, The	**2010**	76,3%	**2005/06**	78,3%							2,0%
Ghana	**2011**	3,8%	**2006**	3,8%							0,0%
Guinea	**2012**	96,9%	**2005**	95,6%	**1999**	98,6%					3,0%
Guinea-Bissau	**2010**	49,8%	**2006**	44,5%							5,5%
Iraq	**2011**	8,1%									0,0%
Kenya	**2008/09**	27,1%	**2003**	32,2%	**1998**	37,6%					10,5%
Liberia	**2013**	55,5%	**2007**	58,2%							8,4%
Mali	**2012/13**	91,4%	**2006**	85,2%	**2001**	91,6%	**1995/96**	93,7%			8,5%
Mauritania	**2011**	69,4%	**2007**	72,2%	**2000/01**	71,3%					2,8%
Niger	**2012**	2,0%	**2006**	2,2%	**1998**	4,5%					2,5%
Nigeria	**2013**	24,8%	**2008**	29,6%	**2007**	26,0%	**2003**	19,0%	**1999**	25,1%	10,6%
Senegal	**2014**	24,7%	**2010/11**	25,7%	**2005**	28,2%					3,5%
Sierra Leone	**2013**	89,6%	**2010**	88,3%	**2008**	91,3%	**2005**	94,0%			4,4%
Somalia	**2006**	97,9%									0,0%
Sudan^b^	**2010**	87,6%	**2000**	Unspecified	**1989/90**	89,2%					1,2%
Tanzania	**2010**	14,6%	**2004/05**	14,6%	**1996**	17,9%					3,3%
Togo	**2013/14**	4,7%	**2010**	3,9%	**2006**	5,8%					1,9%
Uganda	**2011**	1,4%	**2006**	0,6%							0,8%
Yemen^c^	**2012/13**	18,5%	**2003**	38,2%	**1997**	22,6%					19,7%

Data stemming for MICS surveys are underlined. Data collected via DHS surveys are not underlined in the table

This table presents the FGM prevalence (age 15–49) in countries of origin according to DHS and MICS surveys (year of publication until May 2015, national % FGM) and the difference between the minimum and maximum over all surveys

^**a**^Eritrean Population and Health Survey (2010)

^b^Sudan Household Health Survey SHHSII (2010); report of 2000 did not give a total fgm prevalence

^c^National Health and Demographic Survey (2003; 2012–13)

Thirdly, several prevalence studies [34, 37, 41, 43] have also mentioned the need to include information about ethnicity in the countries of origin. Indeed, for low-prevalence countries (e.g. Uganda) or high-prevalence countries (e.g. Somalia), information about ethnical background is not essential since virtually none or almost all ethnicities will be subjected to FGM [75], but in countries such as Senegal (with an FGM prevalence rate of 24.7% [76]), Liberia (49,8%) [72] or Mauritania (69.6%) [77] there is a mix of ethnicities with some of them applying and others defying the practice. For example, data from Senegal [76] show that 24.7% of the women between 15 and 49 have undergone FGM, but ethnically speaking, the prevalence varies from 1.3% (among the Wolof) to 64.4% (among the Mandingue). However, information about ethnical background is not sufficient as variations within ethnic groups equally exist. For example, the prevalence of FGM among the Fulani is at 12.7% in Cameroon [78], 41.2% in Benin [79], 83.9% in Burkina Faso [74] and 99.5% in Guinea [80].^5^ On the other hand, the FGM prevalence of Somalians in Kenya is 97.6%, closer to the national average of Somalia (97.9%) than to that of Kenya (27.1%) [63]. As a result, only the combination of ethnicity and country of origin might result in more reliable prevalence estimations.

Fourthly, indirect estimations are also problematic when it comes to the inclusion of daughters from women who are originating from countries where FGM is practiced into prevalence estimations. The terms of ‘first generation’ and ‘second generation’ are used in an incoherent way, which results in operational confusion. It is therefore important to take into consideration that the term ‘generation’ in demography is used differently than in the migration context. A mother and her daughter can be both first generation in terms of migration when they arrive together in the EU, but in demographical terms the mother is always first generation whereas the daughter is always second generation, no matter where the daughter is born. Or put differently, the daughter can be a first- or second-generation migrant, but she is always second generation in the demographical sense of the word. This distinction is not always very clear in the existing prevalence studies. For example, Farina and Ortensi [37] explain in the introduction of their paper that their study concerns an ‘estimation of the prevalence of FGM among first generation migrants’. When they mention ‘*second generation girls’* they mean *daughters who have migrated to the EU* and not those who are born here. However, other studies focus mainly on ‘generation’ in a migration context when talking about daughters. For instance, “Girls born in Belgium from a mother having adopted the Belgian nationality are registered as Belgian, and hence it is impossible to find the second generation girls.” [41]. Here ‘*second generation girls’* are *daughters who are born in the EU*. The same terminology is used by Exterkate [43]: “2^nd^ generation: a person who is born in the Netherlands with at least one parent born abroad.” Since ‘generation’ is a term that is equally important both in demography and migration, it is difficult to find a way out for this problem. A possible solution could be to talk about ‘descendants’ when it concerns daughters of women originating from countries where FGM is practiced and to use ‘generations’ when talking about migration.

Fifthly, another area of concern with indirect estimations is how to take the impact of migration into account. Within the first generation, migration selectivity may play a role. Women who migrate might not be representative for the women in the countries of origin. Some studies suggest that migrants tend to be more educated compared to non-migrants [48, 81, 82] and that they are often wealthier, younger and more urbanized than the overall national profile [48, 61, 82, 83]. Moreover, once women have migrated, their views on FGM might have changed as a result of prevention campaigns or because of fear for a strict legislation [43, 75, 84]. Living in a migration context can also constitute an enabling environment to resist social pressure to perform FGM [50, 85]. Or they adopt immediately the habits of the host country, like the Ethiopian Jews who gave up on FGM directly upon arrival in Israel without any signs of distress or nostalgia [86]. Legislation and prevention campaigns may thus influence the FGM status of descendants of first generation migrants and of first migration generation girls who left their country of origin before the age of FGM. Finally, women can migrate because they want to flee the risk of FGM, for themselves or for their daughters. In some countries in the EU such as Belgium, FGM is considered a form of prosecution and therefore recognized as grounds to be granted refugee status [26]. But also the opposite has been mentioned: Somalian Bantu refugees forced their daughters to FGM shortly before their resettlement to the us because they knew that the practice was prohibited by the us legislation [87]. All factors impose problems when applying the extrapolation-of-fgm-countries-prevalence-data-method.

As a consequence, the time between arrival in the country of destination and time of measuring could be considered crucial in the estimation of FGM prevalence. The idea that the longer migrants are exposed to a society where the norms are opposing FGM, the more they will be reluctant to perform FGM. However, in their study about mother to daughter transmission of FGM, Farina and Ortensi conclude: “The number of years elapsed since migration is not a good variable to directly explain the impact of migration on a girl’s risk of undergoing FGM because this information does not tell us anything about the family’s interactions within their social environment, which could lead to either integration or isolation” [48].

Finally, several FGM prevalence studies [34, 41, 43] have tried to incorporate information about applicants for international protection. When estimating the prevalence of FGM in the EU, it remains to be seen if it is important to consider the proportion of applicants for international protection to the total group of migrants from the 29 countries of origin. The absolute number of applicants for international protection from countries where FGM is practiced increased substantially between 2011 and 2014 (and correspondingly also the number of girls and women that have potentially undergone FGM). In 2014, there were 25,980 girls and women from one of the 29 countries where FGM is practiced who applied for international protection in EU (of which an estimated number of 15,947 have been subjected to FGM) [28].

The estimated number of new applicants for international protection who have been subjected to FGM is low compared to the estimated number of potentially affected girls and women who are already registered in the official administrative records of the country of destination. This is illustrated by Table 3.

**Table 3:** Estimated number of new applicants for international protection (potentially) subjected to FGM

	Year of publication	Estimated number of girls and women potentially subjected to FGM [26]	Estimated number of female applicants (14–64) potentially subjected to FGM in 2014 (EUrostat, 2014 - https://ec.europa.eu/CensusHub2)	% of new applicants compared to the existing population of girls and women subjected to FGM
Belgium	2011	6260	626	10%
France	2007	61,000	1928	3%
Germany	2007	19,000	4269	22%
Hungary	2012	350	121	35%
Ireland	2011	3170	12	0%
Italy	2009	35,000	763	2%
UK	2007	65,790	1254	2%
The Netherlands	2013	28,000	968	3%

Proportion (%) of the estimated number of female applicants in 2014 from countries where FGM is practiced (Eurostat, 2014) to the estimated number of girls and women who have potentially undergone FGM who were already registered in the official administrative records of the country of destination (EIGE, 2013)

Particularly for high prevalence countries in the eu, the number of applicants for international protection from one of the 29 countries where FGM is practiced, compared to the existing population remains marginally low between 0 and 3%. Only for Hungary (with a very low absolute prevalence number), Germany (where the estimation dates back from 2007) and Belgium, the proportion of new applicants for international protection to the existing population is considerable.

Given the low number of applicants for international protection on the total number of migrants from the 29 countries where FGM has been documented, the importance of including applicants for international protection as a separate group in a prevalence study is therefore limited.

### Strengths and limitations of direct estimation methods

Many of the limitations of the indirect methods could be controlled in direct estimations, e.g. ethnicity, impact of migration, legal status of the respondent, fluctuation of prevalence figures in the countries of origin, or the confusion between first and second generation in a migration and generational context. However, direct estimations have also limitations.

Firstly, one of the main challenges that direct estimation methods have to deal with is the fact that FGM is a sensitive topic to talk about [85]. Respondents might be reluctant to provide answers out of fear of repercussions when the practices has been outlawed [75] or out of fear of stigmatization when norms in the country of destination regarding FGM are different than those of countries of origin [30].

However, the direct methods allow on the other hand to inquire about sensitive information in less straight forward manners. Another way to introduce the subject for example, is through the use of local terminology. In some countries of origin such as Liberia, the researchers of the DHS decided to eliminate any direct reference towards FGM because of the sensitivity of the topic. Instead, the question was introduced indirectly and respondents were asked whether they had been initiated into a women’s secret society such as the Sande [72, 88]. The results were used as a proxy for the estimation of the number of women who were subjected to FGM since membership of the secret society requires that a woman undergoes FGM.

If interviewers are not thoroughly selected, trained and monitored, this might have a considerable impact on the interview [30] and thus on the direct estimation. Using same sex interviewers and facilitators from the same ethnic background as the participants in focus groups are equally important [89]. Providing socially desirable answers is a known weakness in conducting interviews and focus group discussions that might have an impact on the outcome.

However, if these sensitivities are properly addressed, FGM is a subject that can be talked about if it is done by the right person in a respectful, non-offensive way. As a consequence, respondents are willing to address the issue with little or no reluctance and their answers will be reliable. In their direct estimation, Andro et al. [30] reported an unexpectedly low number of drop-outs or refusals, which has been confirmed by other research such as EIGE [85] and Exterkate and de Jager [89]. Other researchers even noted an expressed need to break the silence around FGM in respondents through the participation in the research of the respondents [30, 85].

Secondly, direct estimations rely on the self-reported status of FGM by the interviewee. The reliability of this self-reporting has however been questioned [90–92]. Andro et al. [30] highlighted this discrepancy when both a survey and a clinical examination by a trained gynaecologist of the genital area were performed. The answers of women did not always match with the FGM-status reported by the gynaecologist, but at the same time, doctors did not seem to be experts in distinguishing the different types of FGM^6^ either [30, 49]. Especially when it comes to the type 1 or some forms of Type IV. However, the use of self-reported data does not necessarily lead to better or worse results compared to data from medical examinations and that there is sufficient ground for calculating FGM prevalence based on self-report studies [37, 75, 90].

## Limitations

The limited amount of national FGM prevalence studies in the EU had a significant influence on the SWOT-analysis. Although this study did not cover studies conducted from August 2015 onwards, the lessons learned and recommendations resulting from the FGM-PREV project are still relevant [28]. We searched for other papers published after August 2015 which referred to our proposed method. This showed that it has been used or referred to three times: the FGM-PREV study resulted in a highly cited paper on the estimates of first-generation women and girls with female genital mutilation in the European Union, Norway and Switzerland [93], in another paper on FGM estimates in first-generation women and girls in Italy [94] and in a study on the practice of FGM across the world [18].

Additional research is necessary to further evaluate the proposed FGM prevalence estimation method within this study. Designing a common composition and methodology to estimate FGM prevalence and number of girls and women subjected to FGM in the EU, should be an evaluative process consisting of repeated SWOT analyses and lessons learned in order to fine-tune the methodology and make it applicable throughout all the EU member states, with sufficient flexibility to be adapted to the actual and present-day circumstances regarding migration flows and legislation within a given EU member state.

## Conclusions

Both indirect and direct methods to estimate the number of girls and women subjected to FGM in the EU and the FGM prevalence in the EU have strengths, weaknesses, opportunities and treats. The choice for a direct or indirect method will depend on available financial means and the purpose for the estimation.

Direct and indirect estimations of FGM prevalence and the number of girls and women subjected to FGM are more accurate if they consider regional origin, and not merely nationality, as well as ethnicity in the countries where FGM is documented rather than geographical distribution. The accuracy increases even more when the estimations are based on a combination of ethnicity and country of origin. Moreover, the impact of migration should equally be considered. Applicants for international protection and undocumented migrants do not have a different FGM-profile compared to other migrants originating from countries where FGM is documented, and their proportion is considered to be low when calculating the prevalence. Exceptions can be made for new groups of applicants for international protection who were previously not present in the country of destination.

Further, if addressed in an appropriate way by well selected and trained interviewers, women will not be reluctant to disclose their FGM-status, which is crucial for direct FGM estimations. Direct estimations of FGM prevalence does not necessarily require a clinical examination since self-reported data is reliable enough and less intrusive.

Based on these assumptions, we suggest the following approach for striving to a comparable method to estimate FGM prevalence and the number of girls and women subjected to FGM throughout the different EU member states. The first step towards generating comparable data relates to the use of a common composition of FGM prevalence within the migrant population. We would suggest to add daughters of women who originate from countries where FGM is practiced to the definition of prevalence of FGM as defined by EIGE [26]: “The prevalence of FGM in any of the Member States of the EU is defined as the number of women and girls in that country who have undergone FGM at a certain point in time expressed as the proportion of the total number of women living in the country but originating from countries where FGM is practiced, *and their female descendants*.”

As to decide upon the most appropriate methodology for the estimation of FGM prevalence and number of girls and women affect by FGM in the EU, we propose a twofold model based on the initially expected prevalence in a given EU member state. Due to the relatively low number of women who have undergone FGM compared to the general population in the EU, a nationwide randomized sample is not feasible. Moreover, migrants from countries where FGM is documented are not equally distributed over the EU. Some countries (e.g. Italy, France, Germany, The Netherlands) have a high prevalence of women who might have undergone FGM, while the prevalence in other countries might be very low (e.g. Latvia, Estonia, Hungary, Czech Republic). Therefore, these two situations need a different approach.

For countries with a low expected prevalence of women who have undergone FGM, the indirect method, based on a triangulation of population data and/or census data and data from DHS and mics, the so-called ‘extrapolation-of-fgm-countries-prevalence-data-method’, will give a good enough estimation of the number of women who have potentially undergone FGM. Depending on the situation, the extrapolation could be done based on the raw FGM percentages as mentioned in the DHS, or a correction based on age, wealth, level of education and urbanization [48, 82] could be applied or it might be possible to opt for an interval with a minimum and a maximum [30, 43]. For prevention measure, policy making and training of health specialists these figures will give a good enough indication of the needs regarding women who might have undergone FGM. An estimated number for example is sufficient to get an overall idea of the regions where girls and women who may be subjected to FGM live.

For countries with a high expected prevalence of women who have undergone FGM, this method could be the starting point to estimate the prevalence of girls and women who have undergone FGM. In many countries (e.g. Belgium, The Netherlands, France, Italy, uk) this has been done already. However, only a direct estimation will provide with more accurate information. Therefore we recommend to combine both a direct and indirect estimation method and to use a sample design as developed by the FGM-PREV project [28]. Given the low number of applicants for international protection and undocumented migrants on the total number of migrants from the 29 countries where FGM has been documented, and the fact that there is no reason to believe that the profile of the applicants for international protection and undocumented migrants differs significantly from migrants who have a residence status, the importance of including applicants for international protection and undocumented migrants as a separate group in a prevalence study should be well balanced against the practical challenges to find them.

Within the direct FGM prevalence estimation part of this approach, we consider a careful selection and training of interviewers (e.g. ethnicity, language, attitudes towards FGM) and a questionnaire that considers these sensibilities as indispensable given the sensitivity of the topic. Since the use of self-reported data does not necessarily lead to better or worse results compared to data from medical examinations and the ethical implications of clinical examinations, there is sufficient ground to estimate FGM prevalence through surveys based only on self-reported data.

The surveys should be developed in a way that they can be used to perform longitudinal research, i.e. to allow the same survey to be conducted at regular intervals in order to measure changes in profiles of migrants and their attitudes towards FGM. Age categories in surveys should be expanded to girls under 15 (even indirectly by asking the mother) and women above 50 and surveys will have to make the distinction between ‘descendants’ when it concerns mothers and daughters and to use ‘generations’ when talking about migration. Both aspects are equally important.

Furthermore, the surveys will allow to compare the results with the outcome of the indirect estimations through the extrapolation-of-fgm-countries-prevalence-data-method. Therefore, other questions that are not directly relevant for a survey (e.g. about ethnicity or age of FGM) could be included in the questionnaire. It allows the corrections that are applied to the extrapolation-of-fgm-countries-prevalence-data-method to be more precise. Since the nature of migration is changing so rapidly, in high FGM prevalence countries surveys could be held every 3 to 5 years, whereas the extrapolation-of-fgm-countries-prevalence-data-method could be applied every year to the existing data provided by the national statistical offices and corrected by the outcome of the surveys.

## Data Availability

The datasets used and/or analysed during the current study are available from the corresponding author on reasonable request.

## Abbreviations

DHS: Demographic health survey
EIGE: European Institute for Gender Equality
EU: European Union
EU-27: European Union in the period of 2007–2013 when it consisted of 27 countries
FGM: Female genital mutilation
FGM-PREV: Research project: “FGM-PREV: Towards a better estimation of prevalence of female genital mutilation in the European Union”
MICS: Multiple Indicator Cluster Surveys
SWOT: Strengths, weaknesses, opportunities & threats
UK: United Kingdom
UN: United Nations
UNICEF: United Nations Children’s Fund

## Footnotes

1 Additionally, surveys were conducted in Indonesia in 2013 but at the time of this study (2015) this data was not yet available. At the time of the study FGM was thus only well documented in Africa and the Middle East.

2 Despite the independence of South Sudan anno 2011, there is no DHS survey for South Sudan specifically.

3 For more information see Leye, E., et al. (2017). Estimating FGM Prevalence in Europe. Findings of a Pilot Study. Research Report. Ghent, UGent-ICRH.

4 Some studies such as EIGE (26. EIGE: Female Genital Mutilation in the European Union and Croatia. Lithuania: European Union; 2013.) refer to the method as the ‘extrapolation-of-African-prevalence-data-method’. However, since there are countries outside of Africa such as Yemen and Iraq where fgm is practiced, this report will use the term ‘extrapolation-of-fgm-countries-prevalence-data-method’.

5 The Fulani are registered among different names according to the country: in Cameroon ‘Arabe-Choa/Peulh/Maoussa/Kanuri’ [77], in Benin ‘Peulh et apparentés’ [78] and in Burkina Faso ‘Fulfuldé/Peul’ [79].

6 The who distinguishes four different types of fgm [14]: Type I: Partial or total removal of the clitoris and/or the prepuce (clitoridectomy). Type II: Partial or total removal of the clitoris and the labia minora, with or without excision of the labia majora (excision). Type III: Narrowing of the vaginal orifice with creation of a covering seal by cutting and appositioning the inner or outer labia, with or without excision of the clitoris (infibulation). Type IV: All other harmful procedures to the female genitalia for non-medical purposes.
